# Deep learning for drug‐drug interaction prediction: A comprehensive review

**DOI:** 10.1002/qub2.32

**Published:** 2024-02-13

**Authors:** Xinyue Li, Zhankun Xiong, Wen Zhang, Shichao Liu

**Affiliations:** ^1^ College of Informatics Huazhong Agricultural University Wuhan China

**Keywords:** deep learning, drug‐drug interactions, graph neural network, knowledge graph, multimodal deep learning, neural network

## Abstract

The prediction of drug‐drug interactions (DDIs) is a crucial task for drug safety research, and identifying potential DDIs helps us to explore the mechanism behind combinatorial therapy. Traditional wet chemical experiments for DDI are cumbersome and time‐consuming, and are too small in scale, limiting the efficiency of DDI predictions. Therefore, it is particularly crucial to develop improved computational methods for detecting drug interactions. With the development of deep learning, several computational models based on deep learning have been proposed for DDI prediction. In this review, we summarized the high‐quality DDI prediction methods based on deep learning in recent years, and divided them into four categories: neural network‐based methods, graph neural network‐based methods, knowledge graph‐based methods, and multimodal‐based methods. Furthermore, we discuss the challenges of existing methods and future potential perspectives. This review reveals that deep learning can significantly improve DDI prediction performance compared to traditional machine learning. Deep learning models can scale to large‐scale datasets and accept multiple data types as input, thus making DDI predictions more efficient and accurate.

## 1 INTRODUCTION

Concomitant administration of multiple drugs may enhance or diminish their effects [1], or even lead to adverse drug reactions [2] and increased morbidity and mortality in patients [3]. These interactions are called drug‐drug interactions (DDIs). In order to prevent harmful DDIs, DDI prediction is critical and can provide important information for medical researchers to refer to. With the increasing popularity and improvement of deep learning and the expansion of drug databases, a large number of deep learning‐based DDI prediction methods have been proposed and become more diverse.

**FIGURE 1:**
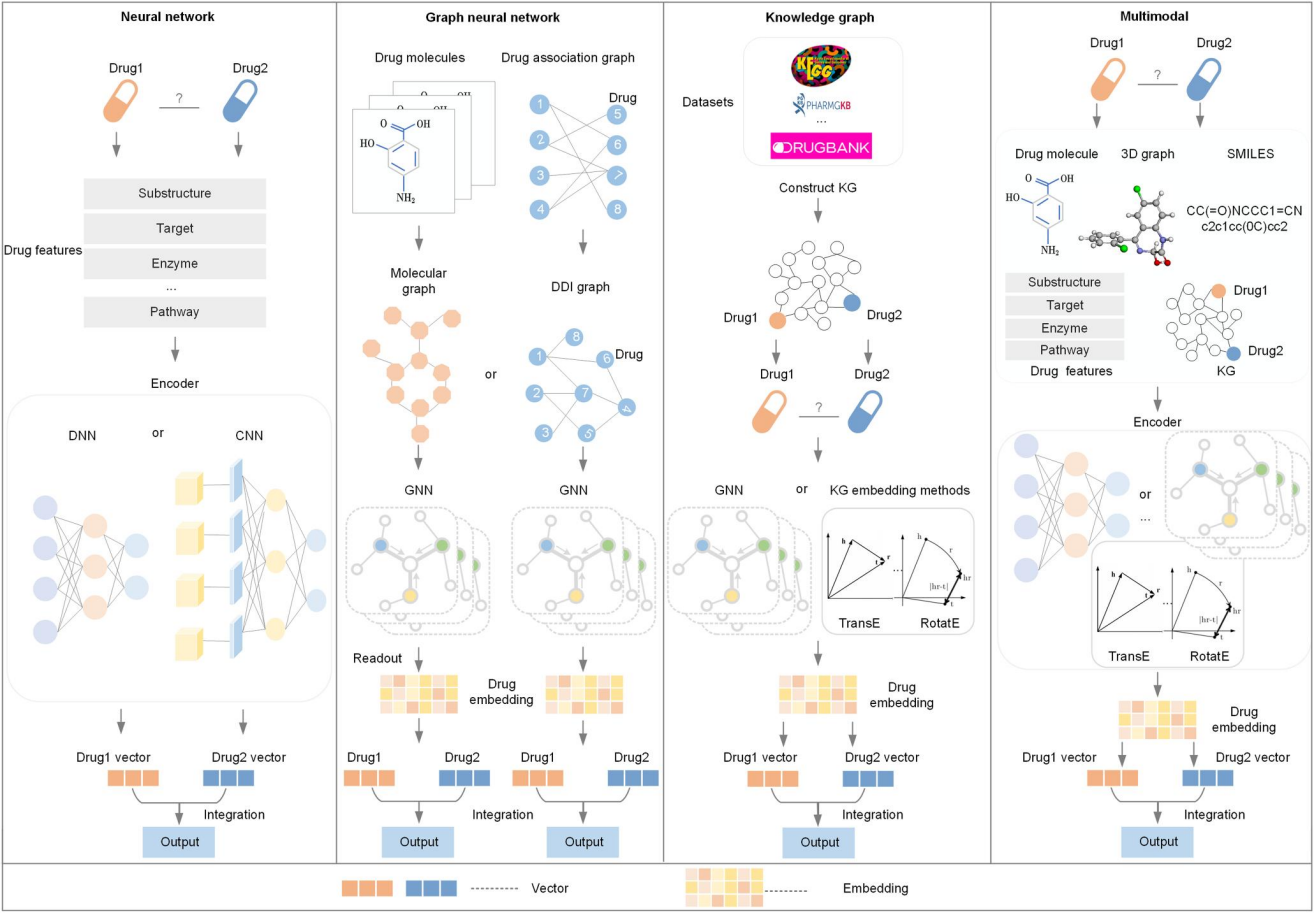
The overall workflow of four kinds of deep learning methods for DDIs prediction.

The majority of deep learning‐based DDI prediction methods are based on neural networks, which is the most commonly used method for deep learning. In recent years, graph neural networks (GNNs), which have shown its strong power in directly modeling natural graph structure data, have been widely utilized in DDI prediction [4, 5, 6, 7]. Researchers usually construct drug molecular graphs or drug association graphs from drug molecular structures or drug‐related associations and use GNNs to learn drug representations from these graphs for DDI prediction without feature engineering or expert knowledge preprocessing. Considering that DDIs are usually driven by complicated biomedical mechanisms and the common drug association graphs may not well model the large number of biological entities and multiple complex associations in biomedical networks, researchers noticed the Knowledge graphs (KGs) can contain a large number of entities and associations. Researchers collect abundant biomedical entities and associations related to DDIs, construct KGs, and utilize KG embedding methods or GNNs to learn global or local information [8] of drugs for DDI prediction. Although the above methods have good performances in predicting DDI, some researchers think that considering multimodal data (such as multi‐types of drug features, molecular graphs, DDI association graphs, KGs.) can improve the performances of DDI prediction. Therefore, researchers propose some multimodal‐based methods which can integrate multimodal data of drugs and learn more comprehensive drug representations for more accurate DDI prediction. The overall workflow of these four kinds of deep learning methods for DDIs prediction is shown in Figure 1.

This article provides a comprehensive review of the recent advances in DDI prediction in the field of deep learning, and mainly discusses deep learning methods for DDI prediction without considering DDI extraction. In this paper, the methods of DDI prediction in deep learning are divided into four categories: Section 2 is about neural network‐based methods, Section 3 is about GNN‐based methods, Section 4 is about KG‐based methods, and Section 5 is about multimodal‐based methods. The main variables and symbols used in this paper are listed in Table 1.

**TABLE 1:** The main variables and symbols used throughout the paper.

Variable	Description
Upper‐case letter	Matrix
Lower‐case bold letter	Vector
Lower‐case non‐bolded letter	Scalar quantity
T	The triplet <v,r,u>
‖	Concatenation
⊗	The cross‐product operator
⊙	The Hadamard product
°	The element‐wise product operator
σ	The sigmoid function
∗	The convolution operator
ϕ	The non‐linear element wise activation function

## 2 NEURAL NETWORK‐BASED METHODS

Using neural network structure for modeling is the most common method in deep learning methods, as well as the DDIs prediction methods with deep learning. We summarize these methods and show them in Table 2. Traditional neural networks mimic biological neural networks in nature and consist of many neurons connected in a complex way, generally consisting of an input layer, hidden layers, and an output layer. In DDI prediction, we usually use complex transformations or feature extraction on drugs before feeding them into a neural network. Convolutional neural network (CNN) is a feed‐forward neural network that uses convolution kernels to extract suitable local features [9], and after several convolutional layers, nonlinear activation functions and pooling layers, the probability of DDI is finally computed using fully connected layers and softmax function. These methods are described in detail below.

**TABLE 2:** DDI prediction based on neural network.

Taxonomy	Model	Techniques	Reference
DNN‐based methods	NDD	Integrated similarity and deep neural network (DNN)	[10]
	DeepDDI	Structural similarity profile (SSP) and DNN	[11]
	Shtar et al.	Matrix factorization and artificial neural networks	[12]
	$D^{3}I$	Encoder and neural network	[13]
	STNN‐DDI	Tensor neural network	[14]
	CASTER	Chemical sequential pattern mining algorithm (SPM) algorithm, autoencoder, and deep dictionary representation.	[15]
CNN‐based methods	MGP‐DR	Transformer encoder (BERT).	[16]
	CNN‐DDI	Multi‐type feature fusion and convolution neural network	[17]
	META‐DDIE	Few‐shot learning, sequential pattern mining algorithm (SPM), autoencoder, and CNN	[18]
	Molormer	Siamese network, lightweight self‐attention and CNN	[19]

### 2.1 DNN (deep neural network)‐based methods

DNNs are excellent at classification without the need for feature extraction, there are many methods that use DNN to classify the type of DDI for a given drug pair [10, 11, 12]. For example, DNN such as autoencoders can learn to map data from high dimensional space to lower dimensional space [20].

#### 2.1.1 Drug‐drug interaction prediction by neural network using integrated similarity

Rohani et al. [10] combined similarity selection and fusion methods with neural networks to propose an integrated similarity neural network‐based method NDD for drug‐drug interaction prediction. NDD collects data on the similarity of various drugs from five different datasets, then obtains multiple similarity matrices based on different similarity types and Gaussian Interaction Profile (GIP), then NDD selects the high information and low redundancy similarity matrices and integrates them by the nonlinear similarity fusion method SNF to obtain an m×m integrated similarity matrix. Subsequently, the corresponding rows of the integrated similarity matrix of each drug pair are concatenated and fed into a two‐layer neural network. A dropout layer is used after each neural network layer to prevent overfitting. Finally, the ReLU [21] activation function is used in the output layer as a sigmoid function to predict the probability of DDIs. Similarity selection uses the similarity selection heuristic process of Olayan et al. [22]. The advantage of NDD is that by using similarity‐based selection and fusion computational methods, NDD can significantly improve the performance of predicting new DDI on datasets with high complexity and requiring high‐order features.

#### 2.1.2 Combination of structural similarity profile (SSP) and DNN

Existing methods only predict the probability of DDI, or require detailed drug information, to address these issues, Ryu et al. [11] proposed DeepDDI, which consists of structural similarity profile (SSP) and DNN. DeepDDI uses only drug names and structural information of drug pairs as input to accurately predict DDI types. The SSP is a vector representation obtained by computing the chemical structure similarity between drugs and drugs. DNN is a multi‐label model with 8 hidden layers of a simple neural network. The input to the model is SMILES sequences of the chemical structure of drug pairs, and the chemical structure similarity between two drugs is calculated to obtain the SSP. Then the feature representations of the two drugs are connected and the resulting feature vectors are fed to DNN for prediction. The advantage of DeepDDI is that it can accurately predict the type of DDI for a given drug pair and drug‐food composition pair, using only the name and chemical structure as input.

#### 2.1.3 Adjacency matrix factorization (AMF) and adjacency matrix factorization with propagation (AMFP)

To address the fact that similarity‐based DDI prediction is still inadequate in terms of performance, Shtar et al. [12] considered DDI prediction problem as the link prediction problem and proposed two new methods on artificial neural networks: AMF and AMFP. They used XGBoost [23] to train an integrated classifier. In AMF, the embedding layers act as latent factors and biases are shared among the input nodes. The Adam algorithm is used to optimize the element‐wise multiplication and partial order. Based on AMF, the adjacency matrix factorization with propagation AMFP is proposed. Potential factors and propagation factors play an essential role in AMFP. The latent factor is controlled by the propagation factor and propagates to the drug with which it interacts. Each drug shares the latent factor with its neighbor nodes. The advantage of this method is that it uses ensemble‐based classifiers to improve performance by combining various predictors.

#### 2.1.4 Cardinality‐invariant and order‐invariant high‐order DDI prediction

Emerging high‐order DDI research methods only focus on discovering high‐order DDIs by effectively mining frequent drug combinations. To solve this problem, Ning et al. [13] proposed a deep learning model $D^{3}I$ for higher‐order DDIs prediction by using an encoder and an aggregator, which is base‐invariant and order‐invariant and can predict the type of ADR produced by any drug combination of any number of drugs. Firstly, Ning et al. used an *n*‐layer neural network as an encoder, that is $g_{e}\left(f\right)=g_{n_{e}}\left(\text{⋯}\left(g_{2}\left(g_{1}\left(f\right)\right)\right)\right)$, the encoder encodes each drug feature as a drug embedding. The aggregator aggregates the multiple drug embeddings obtained from the encoder. Three aggregation mechanisms are used in the aggregator: max pooling, average pooling and attention aggregation, their process can be calculated as follows, respectively:

$$e_{D}=\max\left(e\left(D\right)\right)=\left[\max_{\forall i}\left\{e_{i,1}\right\},\max_{\forall i}\left\{e_{i,2}\right\},...,\max_{\forall i}\left\{e_{i,k}\right\}\right],$$

$$e_{D}=\text{mean}\left(e\left(D\right)\right)=\left[\underset{\forall i}{\text{avg}}\left\{e_{i,1}\right\},\underset{\forall i}{\text{avg}}\left\{e_{i,2}\right\},...,\underset{\forall i}{\text{avg}}\left\{e_{i,k}\right\}\right],$$

$$e_{D}=\sum_{i=1}^{n}a_{i}e_{i},$$

where $e_{i}$ is an embedding for each drug $d_{i}$. $E\left(D\right)=\left[e_{1},e_{2},\text{…},e_{n}\right]$ is the embedding of all drugs, and is a n×k matrix. $a_{i}$ is a weight on $e_{i}$. Finally, the aggregated drug combination is fed into the predictor, and the tanh activation function is used in a fully connected layer to obtain the final ADR score. The authors believe that although it shows excellent performance in high‐order DDI prediction, it only uses different drug features separately, but multidrug feature fusion can achieve even better performance.

#### 2.1.5 Substructure‐aware tensor neural network to predict DDIs

When making DDI predictions, models are often required to have the ability to make inductive predictions for practical reasons, but many network and matrix factorization based methods do not meet this requirement, besides many current methods are also lacking in interpretation. To solve these problems, Yu et al. [14] proposed a substructure‐aware tensor neural network STNN‐DDI. The model learns a three‐dimensional tensor of <substructure, substructure, interaction type>. The substructure‐substructure interaction space (SSI space) is composed of the 3D substructure aware tensor and the fully connected neural network. STNN‐DDI first inputs the triplet and then randomly initializes all substructures and interaction embeddings. These substructures and interaction embeddings constitute factor matrices A and C in rows, separately. Finally, the fully connected neural network is constructed for calculation according to this equation:

$${\hat{P}}_{pqk}^{d}=\left[\left(e_{q}^{T}\cdot A\right)⊙\left(e_{p}^{T}\cdot A\right)⊙\left(v_{k}^{T}\cdot C\right)\right]\cdot W_{\lambda }+b,$$

where $W_{\lambda }$ is the weight of each rank‐one tensor in tensor reconstruction, b is added for enhancing the robustness of STNN‐DDI, $e_{q}$ is the fingerprint of drug $d_{q}$, $v_{k}$ is the embedding of the relation $l_{k}$. The advantage of STN‐DDI is its good interpretability. It can reveal both important substructural pairs related to DDI types in drugs and major interaction type‐specific substructural pairs in a given DDI.

#### 2.1.6 A chemical substructure representation framework for DDI prediction

Existing methods for DDI prediction lack drug labeling design specifically for DDI prediction and also suffer from limited generalization performance and poor interpretability. In response to the above issues, Huang et al. [15] proposed a DDI prediction model CASTER based on chemical substructure representation. They parameterized the weights and biases in the encoder via a neural network (NN): $z_{t}=W_{e}x_{t}+b_{e}$, and another NN framework is defined in the decoder, using the decoder reconstruction functional representation ${\hat{x}}_{t}=\text{sigmoid}\left(W_{d}+z_{t}+b_{d}\right)$. CASTER extracts frequent substructures from the molecular database by sequence pattern mining (SPM) algorithm [24]. Subsequently generates a function representation *x* for each input pair. The function representation is then embedded into the latent space by minimizing the reconstruction loss to obtain the latent feature vector *z*. Meanwhile, the function representation of each frequent substructure is also embedded into the latent space and a dictionary entry is generated. The advantage of CASTER is that it can provide more accurate and interpretable DDI predictions.

#### 2.1.7 Molecular graph pretraining framework for drug pair representation learning used neural network

Existing methods are limited by redundant drug feature collection, small amount of labeled data, and low model generalization ability. Meanwhile, multi‐drug representative learning also lacks unique methods. To solve these problems, Yu et al. [16] designed a pre‐training framework MGP‐DR specifically for drug pair representation learning. This framework is based on pre‐trained models and multi‐drug representation learning of molecular graphs for DDI prediction by integrating a large amount of unlabeled drug molecule graph information and target information. First, two strategies for multi‐molecular graph representation are designed. In Strategy 1, the supernode is connected to each atom in both drugs. In Strategy 2, supernode 1 (SUP1) is connected to all atoms in drug A and supernode 2 (SUP2) is connected to all atoms in drug B. The hidden structure and information in drug pairs are then mined using two pre‐training tasks: masking atomic prediction and $S_{\text{AB}}$ fraction prediction. The masking operation was performed on the entire drug‐pair molecular graph, with 15% of the atoms selected at random. To better characterize the drug‐target overlap, the average shortest distance between target pairs is compared with the average shortest distance between target proteins within each drug, $<d_{\text{AA}}>$ and $<d_{\text{BB}}>$ to obtain $S_{\text{AB}}$:

$$S_{\text{AB}}=<d_{\text{AB}}>-\left(<d_{\text{AA}}>+<d_{\text{BB}}>\right)/2.$$

If $S_{\text{AB}}<0$, the two drug‐targets overlap, otherwise topologically separated. Before pre‐training, two normalization methods min‐max method and the z‐score method were used to preprocess $S_{\text{AB}}$:

$$X^{*}=\left(X-\min\left(X\right)\right)/\left(\max\left(X\right)-\min\left(X\right)\right),\text{and}\;X^{*}=\left(X-\mu \right)/\delta ,$$

where $X^{*}$ represents the normalized result of the X value, min(X) and max(X) are the minimum and maximum values of X, μ is the mean and δ is the variance. In the transformer encoder layer, each atomic token exchanges information with the global attention mechanism and then adds the local attention mechanism based on chemical bonds to the attention score. The final output layer consists of a fully connected neural network that outputs the $S_{AB}$ prediction and MASK prediction results. The advantage of MGP‐DR is that its training strategy can effectively capture potential intra‐drug and inter‐drug associations, but the authors suggest that it would be helpful to use a well‐established multi‐task learning strategy to combine different pre‐training tasks during the training phase.

### 2.2 CNN‐based methods

CNN generally includes several convolutional layers, nonlinear activation functions and pooling layers, etc. CNN can use convolution kernels to extract appropriate local features. At the same time, it can search for repetitive spatial patterns in the data and combine them into high‐level features [25].

#### 2.2.1 Multi‐type feature fusion based on convolution neural network

Since CNN performs better than DNN in feature learning, it can effectively alleviate the degree of overfitting. Zang et al. [17] proposed CNN‐DDI using CNN, considering that their selected features include noise and CNN’s advantage. CNN‐DDI consists of two main parts: a combined feature selection module and a CNN prediction module. The four drug features of drugs are used to select feature vectors in the combined feature selection module, which generates binary vectors. The similarity matrix of the four features is obtained by calculating the correlations between the drugs using the Jaccard similarity. The four resulting feature similarity matrices are subsequently used as input to the prediction module. The prediction module consists of five convolutional layers, two fully connected layers, and one softmax layer. The LeakyReLU activation function is used in each convolutional layer, and a residual block [26] is added between every two CNN layers to form a short connection that can enhance the correlation between multilayer features.

#### 2.2.2 Few‐shot learning based on convolution neural network

To get around the problem of rare events with only a few examples that prevent accurate predictions, Zhang et al. [18] proposed META‐DDIE, a method to predict DDI events using few‐shot learning. The model takes drug pairs and substructures as input, uses the drug‐drug interaction representation module to learn interpretable representations of DDIs, uses the drug‐drug interaction comparison module G to predict whether the representations of two DDIs are similar, and finally predicts DDI events under the minority learning framework. The generated vectors are obtained using an encoder, and then the latent embeddings are decoded using a decoder, which uses a neural network with a sigmoid function. The drug feature vector $f\left(x_{i}\right)$ and $f\left(x_{j}\right)$ obtained from the representation module are fed into the comparison module. ${\hat{y}}_{i,j}$ represents the similarity between $x_{i}$ and $x_{j}$: ${\hat{y}}_{i,j}=G\left(f\left(x_{i}\right),f\left(x_{j}\right)\right)$. The 1D convolutional layer, batch normalization layer, ReLU activation layer and max‐pooling layer are used successively. The similarity of the two drug feature vectors is finally calculated using fully connected layers and softmax functions. With C‐way K‐shot learning, Zhang et al. can obtain the C‐dimension score for each drug‐drug interaction in the query set, then select the drug with the highest score and classify the DDI. META‐DDIE outperforms other methods in terms of predictive power. However, the authors believe that a small percentage of the collected events have only one example, which can only belong to the support set or the query set and therefore cannot be classified, which can be solved by using zero‐distance learning.

#### 2.2.3 Lightweight self‐attention based on the spatial structure of molecular graphs

To address the problem that traditional DDI prediction methods ignore information about the positions of atoms and edges in the spatial structure, Wang et al. [19] proposed a lightweight method based on self‐attention for drug‐drug interaction prediction using CNN with a ProbSparse self‐attention mechanism. The Molormer model takes the 2D structure of drugs as input and encodes the molecular graph using four features related to spatial information. Then a ProbSparse self‐attention block molecular graph encoder based on sparse self‐attention is proposed. It includes three ProbSparse self‐attention blocks and two CNN blocks. CNN is added after the first two self‐attention blocks to extract the feature maps. In addition, the parameters of the neural network are reduced by sharing weights using Siamese network [27]. ProbSparse self‐attention is calculated as follows:

$${\text{Attn}}_{\left(i,j\right)}=\text{Soft}\max\left(\frac{\left(E_{\text{ato}m_{i}}W_{\bar{Q}}\right){\left(E_{\text{ato}m_{i}}W_{K}\right)}^{Τ}}{\sqrt{d}}+E_{\text{edg}e_{\left(i,j\right)}}\right)\left(E_{\text{ato}m_{i}}W_{V}\right),$$

where $E_{\text{ato}m_{i}}$ is the embedding of atom i, $E_{\text{edg}e_{i}}$ is the embedding of edge i, $W_{\bar{Q}},W_{K}$ and $W_{V}$ are the learnable shared weight matrices. Finally, the two feature maps are connected and fed into a decoder consisting of three fully connected layers and softmax functions for dimension and the final DDI prediction. Although Molormer improves in performance, the authors argue that using the two‐dimensional structure of the drug as model input leads to excessive time and memory used to process the data.

## 3 GNN‐BASED METHODS

Due to the power of GNN modeling on graphs in recent years, many works have applied GNN to DDI prediction to learn drug structure information from graphs. GNN uses message passing mechanism [28] to aggregate information from the neighborhood of the central node and update the representation of the node. It generates graph‐level feature vectors by the readout function to aggregate node features. In this paper, GNN‐based methods are classified into two categories according to the type of input data: molecular graph‐based GNN and association graph‐based GNN. Most existing methods are based on molecular graphs, but association graph‐based methods can consider properties beyond the molecular structure of drugs for more comprehensive DDI prediction. We summarize these methods and show them in Table 3.

**TABLE 3:** DDI prediction based on GNN.

Taxonomy	Model	Techniques	Refs.
GNN based on molecular graph	MSAN	GNN and MLP. Drugs are represented as graphs, nodes represent atoms and edges represent chemical bonds between atoms	[5]
	GCN‐BMP	Siamese GCN with attention mechanism, bond‐aware message propagation and HOLE‐style neural network	[4]
	SSI‐DDI	Multiple GAT layers, co‐attention, and SAGPooling	[29]
	GMPNN‐CS	Gated message passing neural network, MLP and co‐attention mechanism	[30]
	SA‐DDI	Message passing network with the substructure attention	[31]
	AMDE	Graph encoder consists of multiple message passing attention network (MPAN) blocks and frequent consecutive subsequence (FCS) algorithm	[7]
GNN based on association graph	GCNMK	Graph convolutional network with multi‐kernel and fully connected hidden layers for DDI prediction. Input a DDI graph, nodes are drugs and edges are DDIs	[6]
	SPARSE	Sparse hypergraph neural network for learning about the potential characterization of drugs, side effects, and interactions. Autoencoder with encoder and decoder. Input a DDI hypergraph, nodes include drug nodes and side effect nodes	[32]
	Decagon	Encoder based on graph convolutional network (GCN) and decoder based on tensor decomposition	[33]

### 3.1 Molecular graph‐based GNN

Molecular graph‐based GNN uses the drug molecular graph as model input and can convert drug SMILES sequence representation into a molecular graph by RDKit, where the nodes represent atoms and the edges represent the chemical bonds between two atoms. The graph‐level representation of the drug is learned using multiple GNN layers, and the importance of each substructure for the final DDI prediction can be learned through a common attention mechanism.

#### 3.1.1 Molecular substructure‐aware network based on GCNs

To solve the problem of overfitting and the difficulty of acquiring hand‐engineered domain knowledge, Lu et al. [5] proposed a new DDI prediction method MSAN, using information from the original molecular graph to extract substructures, which takes molecular graphs of two drugs as input. To solve the problem of overfitting, the MSAN‐SD module randomly selects half of the input molecular graphs to be augmented. GNN is used to encode these graphs. In the MSAN‐SE module, a transformer‐like structure is used to obtain the attention scores between the queries and the keys. The attention scores can be formulated as follows:

$$O=\text{RELU}\left(\left(Q+\text{soft}\max\left(\frac{QK^{Τ}}{\sqrt{d}}\right)V\right)W_{O}\right),$$

where Q,K,V denote the queries, the keys, and the values, $W_{O}$ is learnable weight, d is the embedding dimension. The advantage of MSAN is that it increases the diversity of training data and facilitates robust extraction of substructures, in addition to introducing a substructure dropping augmentation.

#### 3.1.2 Bond‐aware message propagation based on graph convolutional networks

To address the problem of relying on drug‐related features that may lead to incurring noisy inductive bias, Wu et al. [4] proposed a GNN model GCN‐BMP with Bond‐aware Message Propagation. The model uses RDKit to extract two types of structural information from molecular graphs: an atom list and a multichannel adjacency matrix. These two types of structural information are fed into Siamese GCN as input. GCN encoder learns the embeddings of individual molecules through an iterative update process and output process, and aggregates atomic‐level embeddings to generate graph‐level embeddings. The update iteration process stacks multiple graph convolution layers to learn the embedding of each node in the graph, two new mechanisms are proposed: Bond‐aware Message Propagation and Highway‐based Update. Bond‐aware Message Propagation can be described as follows:

$$h_{i}^{\left(l\right)}=\sum A_{e_{ij}}^{\left(l\right)}h_{i}^{\left(l-1\right)},$$

where $A_{e_{ij}}^{\left(l\right)}$ is the trainable weight parameters shared by the same type of bond $e_{ij}$ in the l‐th layer. Highway‐based Update can be described as follows:

$$z_{i}^{\left(l\right)}=\tanh\left(W_{z}\left[h_{i}^{\left(l-1\right)};h_{i}^{\left(l\right)}\right]+b_{z}\right),r_{i}^{\left(l\right)}=\sigma \left(W_{r}\left[h_{i}^{\left(l-1\right)};h_{i}^{\left(l\right)}\right]+b_{r}\right),$$

where $h_{i}^{\left(l\right)}$ is the candidate hidden state and $h_{i}^{\left(l-1\right)}$ is the previous hidden state, $W_{z}$ and $W_{r}$ are shared weight parameters, $b_{z}$ and $b_{r}$ are shared bias parameters. The output process introduces attention‐based graph pooling. Finally, the data of the drug pair is fed into a HOLE‐based neural network interaction predictor to predict the presence of interactions between the input drugs. The advantage of GCN‐BMP is that it can capture meaningful data‐driven molecular representation and facilitate DDI prediction tasks. However, the authors believe that the graph convolution operator in prevailing GNNs can only simulate 2D molecular structures, which may overlook some critical 3D structural information.

#### 3.1.3 Substructure‐substructure interactions prediction based on graph attention network

Drug substructures are directly manipulated from the drug molecular graph to extract rich features. However, the order of drugs will change in the training phase of the model, and noise information leakage will occur in the substructure extraction phase, which may affect the performance of the model. To solve this problem, Yu et al. [29] proposed a substructure‐to‐substructure interaction prediction method SSI‐DDI for drug‐drug interaction prediction with GAT networks. The interaction prediction task between two drugs is split into pairwise interactions between their respective substructures. Multiple GAT layers are set. The weights are shared between the GAT layers corresponding to the two drugs. In order to determine the importance of each node in the neighborhood using the attention mechanism, the learnable importance weights are assigned to each node. Then all nodes in the neighborhood are aggregated to update each node. The importance of each node is determined by using SAGPooling. Then the READOUT operation is performed, and the substructure scores of the two drugs are obtained. The importance of each pairwise interaction between the substructures can be calculated using the common attention mechanism:

$$\gamma_{ij}=b^{T}\;\tanh\left(W_{x}g_{x}^{\left(i\right)}+W_{y}g_{y}^{\left(j\right)}\right),i=j=1,...,L,$$

where b is a learnable weight vector, $W_{x}$ and $W_{y}$ are learnable weight matrices, $g_{x}^{\left(i\right)}$ and $g_{y}^{\left(j\right)}$ are all the substructure information of the input drugs $G_{x}$ and $G_{y}$. Finaly, the prediction probability of $G_{x}$ and $G_{y}$ can be calculated as follows:

$$p\left(G_{x},G_{y},r\right)=\sigma \left(\sum_{i}\sum_{j}\gamma_{ij}{g_{x}^{\left(i\right)}}^{T}M_{r}g_{y}^{\left(j\right)}\right),$$

where $M_{r}$ is the learnable matrix. Despite the better performance and interpretability of SSI‐DDI, the authors believe that there is still some noise information leakage in the substructure extraction phase, which may affect the performance.

#### 3.1.4 Combination of gated message passing neural network and prediction module

In previous GNN methods, the hidden representation of nodes in each GNN layer was directly treated as the substructural representation of drugs, without taking into account the prediction of different molecular sizes and shapes, to solve this problem, Yu et al. [30] introduced the gated message passing neural network (GMPNN) and proposed a method GMPNN‐CS, which can directly learn substructures of different sizes and shapes of the molecule. The molecular graphs $G_{x}$ and $G_{y}$ of drugs are used as input, then substructure extraction is performed. The undirected molecular graphs are transformed into directed graphs during extraction, and each node in the graph can be used as a center to collect information about all nodes on the path of that node. Meanwhile, GMPNN is introduced to efficiently compute paths in graphs. Subsequently, each of the generated substructures is linearly transformed, and the correlation between the substructures of the two drugs is learned by combining the substructures of the two drugs in pairs and calculating the substructure attention using the common attention mechanism by MLP. Finally, each substructure undergoes a linear transformation, and the DDI prediction is the sum of the interaction scores between each pair of drugs' substructures. The advantage of GMPNN‐CS is that it learns the substructure of drugs of different sizes and shapes to infer whether a pair of drugs cause DDI or not based on their chemical substructure.

#### 3.1.5 A substructure–substructure interaction module (SSIM) for DDI prediction

Most GNN works treat molecular substructures as fixed‐size. However, the sizes and shapes of chemical substructures are irregular. To address this limitation, Chen et al. [31] proposed two strategies, substructure attention and SSIM, and applied the message passing neural network to construct the SA‐DDI model. The model takes drug pairs as input and sends them to the feed‐forward layer. Subsequently, substructures of arbitrary size and shape are extracted using a message‐passing neural network D‐MPNN with structural attention, which iteratively updates the node features at the k‐th iteration to extract substructures of radius *k*, and computes the substructure attention. Substructure attention is then weighted to obtain size‐adapted molecular substructures. The extracted substructures are then fed into SSIM, which is used to assign a score to each substructure of a drug. The score is determined by the interaction probability with the other drug, thus learning the interaction between substructures. The advantage of SA‐DDI is that it is sensitive to the structural information of drugs and can detect key substructures of DDI.

#### 3.1.6 Attention‐based multidimensional feature encoder

Most deep learning approaches for encoding drug information are somehow inadequate, to alleviate this problem, Song et al. [7] encoded drug features in multiple dimensions and proposed a method AMDE. The multidimensional feature decoder further compresses drug feature vectors and can strongly correlate features with the prediction result. Song et al. take the drug SMILES sequences of two drugs as MADE’s input. Two channels are used to process the input, Rdkit and FCS are respectively used to extract the two‐dimensional atom map features and one‐dimensional sequence features of drugs. They are respectively sent to a 2D feature graph encoder and a 1D feature sequence encoder for encoding. The graph encoder consists of multiple MPAN blocks, the sequence encoder consists of multiple encoder blocks. The message passing process can be described as follows:

$$m_{v}^{\left(k\right)}=A_{t}\left({\text{message}}_{w}^{\left(k\right)},h_{w}^{\left(k\right)}e_{vw}|w\in N\left(v\right)\right),$$

$$\begin{array}{c} A_{k}\left(\text{messag}e_{w}^{\left(k\right)},h_{w}^{\left(k\right)}e_{vw}\right)\\ =\sum_{w\in N\left(v\right)}{\text{message}}_{w}^{\left(k\right)}⊙\frac{\exp\left(f_{NN}^{\left(e_{vw}\right)}\left(h_{w}^{\left(k\right)}\right)\right)}{\sum_{w^{\prime }\in N\left(v\right)}\exp\left(f_{NN}^{\left(e_{vw^{\prime }}\right)}\left(h_{w^{\prime }}^{\left(k\right)}\right)\right)} \end{array},$$

where $f_{NN}$ is feed forward neural network, N(v) is a self‐included neighborhood of the node v, $e_{vw}$ is the edge between node v and node w, $h_{w}^{\left(k\right)}$ is the currently hidden feature. The feature vectors generated by the two encoders are then sent to the multidimensional feature decoder, and generate a high‐dimensional vector via feature blending, which is subsequently decoded to finally obtain the prediction of whether DDI occurs or not. The advantage of AMDE is that it integrates drug features from multiple dimensions to improve the effectiveness of downstream prediction tasks.

### 3.2 Association graph‐based GNN

The association graph relates cause, effect, and related elements according to their interactions, which can help find the cause of the effect. The input of GNN models based on association graphs is generally a DDI graph, which includes relevant information such as drugs and DDIs or side effects.

#### 3.2.1 Graph convolutional network with multi‐kernel

Integrating DDIs with different functions into a single DDI graph may omit some useful information, to address this issue, Wu et al. [6] constructed DDI graphs associated with “increase” and “decrease”, where nodes represent drugs and edges represent DDIs. They input the association matrix of these two DDIs and the drug feature matrix into GCN blocks, respectively. After two GCN layers, the learned feature vectors are connected to form DDI vectors, which are connected with the final drug representation matrix and then feed to the fully connected layer to form a multi‐kernel graph convolution network (GCNMK) to predict DDI types. In the GCN block, the first layer of GCN contains two blocks. The “increased” DDI correlation matrix and drug feature matrix and the “decreased” DDI correlation matrix and drug feature matrix are input into these two blocks, and the linear transformation is performed respectively to obtain the node embedding matrix transmitted in the current block. The propagation rules of the linear transformation are as follows:

$$H_{II}^{I}=F_{I}H_{I}^{O}W_{I}^{O},H_{ID}^{I}=F_{I}H_{I}^{O}W_{I}^{\prime O},H_{DD}^{I}=F_{D}H_{D}^{O}W_{D}^{O}\;and\;H_{DI}^{I}=F_{D}H_{D}^{O}W_{I}^{\prime O},$$

where $H_{II}^{I}$ and $H_{DD}^{I}$ are the node embedding matrices transferring within each block, $H_{ID}^{I}$ and $H_{DI}^{I}$ transferring between the two blocks in layer $l_{1}$, $W_{I}^{O},W_{I}^{\prime O},W_{D}^{O}$ and $W_{D}^{\prime O}$ are the weight matrices. $H^{O}$ is the drug feature matrix, the feature matrix together with the graph $G_{I}$ is marked as $H_{I}^{i}$, whereas the other one is $H_{D}^{i}$, at the i‐th layer of GCNs. The calculation formulas for $F_{I}$ and $F_{D}$ are as follows:

$$F_{I}={\tilde{D}}_{I}^{-\frac{1}{2}}{\tilde{A}}_{I}{\tilde{D}}_{I}^{-\frac{1}{2}},F_{D}={\tilde{D}}_{D}^{-\frac{1}{2}}{\tilde{A}}_{D}{\tilde{D}}_{D}^{-\frac{1}{2}},$$

where ${\tilde{A}}_{I}$ and ${\tilde{A}}_{D}$ are the association matrices of the graph $G_{I}$ and $G_{D}$, ${\tilde{D}}_{I}\left(i,i\right)$ and ${\tilde{D}}_{D}\left(i,i\right)$ are the degree diagonal matrices. The second GCN layer contains a block, it integrates the output of the two blocks in layer $l_{1}$. The activation function is then used to obtain the final drug representation matrix. Before block 4, the DDI information matrix and the final drug representation matrix of the GCN block are first connected through the connection layer. The DDI information matrix is obtained by connecting the two final drug representations in each pair of drugs. However, the authors believe that the removal of drugs without any known DDI during the experiment resulted in the model failing to identify DDI in the cleared drugs. In addition, for the problem mentioned above, SGRL‐DDI [34] introduces Balance theory and Status theory in social networks to reveal the topological patterns between DDIs, which are modeled as signed and directed networks.

#### 3.2.2 A sparse hypergraph neural network for learning multiple types of latent combinations

In reality, side effects may have multiple mechanisms that cannot be represented by a single combination of drug features. Moreover, the DDI data is sparse, to address these issues, Nguyen et al. [32] used DDI hypergraph G=(V,E) as input, which includes drugs and side effects, and proposed a sparse hypergraph neural network for learning multiple potential combinations to accurately predict drug interactions. SPARSE learns latent representations of drugs, side effects, and interactions through a hypergraph neural network with a self‐encoder framework consisting of an encoder and a decoder. In the encoder, the DDI hypergraph and drug node features are encoded into the potential space (H) with drug potential representation and side effect potential representation using message passing hypergraph neural network and coded into the interaction of potential features (B). The decoder reconstructs the DDI hypergraph with the newly predicted hyperedges in H and B. The sparsity of the hypergraph $s_{d}$ and the sparsity of the latent interactions $s_{l}$ are defined respectively as:

$$s_{d}=1-\frac{2\left|E\right|}{\left|V_{D}\right|\left(\left|V_{D}\right|-1\right)\left|V_{S}\right|},s_{l}=1-\frac{2\left|A\right|}{{\left|L_{D}\right|}^{2}\left|L_{S}\right|},$$

where $L_{D}=\left\{1,...,K_{D}\right\}$ and $L_{S}=\left\{1,...,K_{S}\right\}$ are the sets of indices of latent features of drugs and side effects, $K_{D}$ and $K_{S}$ are the numbers of latent features, $V=V_{D}\cup V_{S}$, $E\subset V_{D}\times V_{D}\times V_{S}$, $V_{D}$ is a set of drug nodes, $V_{S}$ is a set of side effect nodes. The advantage of SPARSE is that it improves performance and alleviates the data sparsity problem by using DDI hypergraphs with drug features and sparsity regularization.

#### 3.2.3 Modeling polypharmacy side effects with graph convolutional networks

Most of the previous research methods represent DDIs only by the probability score of the interaction and are unable to predict the type of side effects. To address this issue, Zitnik et al. [33] proposed a method Decagon to model multiple drug side effects by constructing a multimodal graph including protein‐protein interactions, drug‐protein targets, and DDIs, where edges of each type represent different inter‐drug side effects. In Decagon, the multi‐drug side effect problem is regarded as the prediction problem of multi‐type edges on multi‐modal graphs. The model consists of an encoder based on a graph CNN and a decoder based on tensor decomposition. In each layer of the encoder, each node representation is updated by message passing. The single‐layer update formula is as follows:

$$h_{i}^{\left(k+1\right)}=\phi \left(\sum \sum c_{r}^{ij}W_{r}^{\left(k\right)}h_{j}^{\left(k\right)}+c_{r}^{i}h_{i}^{\left(k\right)}\right),$$

where $h_{i}^{\left(k\right)}$ is the hidden state of the node $v_{i}$ in the k‐th layer of the neural network, r is a relation type, $W_{r}^{\left(k\right)}$ is a relation‐type specific parameter matrix, $c_{r}^{ij}$ and $c_{r}^{i}$ are normalization constants. In the decoder, a paired representation of nodes is taken as input, and the probability score for the occurrence of each type of edge is reconstructed from it. The advantage of Decagon is that it automatically learns representations of side effects indicative of co‐occurrence of polypharmacy in patients. Another article by the same author, DeepPurpose [35], also uses the encoder framework to build a framework capable of supporting the various downstream prediction tasks mentioned above.

## 4 KG‐BASED METHODS

A KG is a graph‐structured representation of multi‐relational data, which contains rich semantic information and knowledge facts [36]. KG stores entities and their relations in triplet form (head entity, relation, tail entity), for example, (DRUG1, relation type, DRUG2) where a head entity (DRUG1) is connected to a tail entity (DRUG2) through a predicate relation (relation type). Databases commonly used for DDI prediction, such as DrugBank [37] and KEGG [38], are largely constructed as drug information networks, so they can be described as KGs [39]. More biological association information can be obtained through KG for DDI prediction. Existing KG embedding methods can be classified into three categories: translation‐based methods, tensor factorization‐based methods and neural network‐based methods [40]. We summarize these methods and show them in Table 4.

**TABLE 4:** DDI prediction based on KG.

Taxonomy	Model	Techniques	Refs.
Translation‐based	MUFFIN	TransE, MPNN, CNN‐based network and flatten operation for the local and global features, concatenation operation and a fully connected layer for cross‐level representation,element‐wise product for the scalar‐level representation	[42]
	SumGNN	GNN with attention mechanism	[43]
Tensor factorization‐based	MHRW2Vec‐TBAN	MHRW2Vec, SANA‐ComplEx and TextCNN‐BiLSTM‐attention network (TBAN)	[44]
	RANEDDI	RotatE for learning the multirelational embedding of entity, extracting relation‐aware structure embedding from neighbors, and aggregation operation	[45]
	BioDKG‐DDI	ComplEx‐DURA for obtaining the drug representation vector, SNF for similarity matrix fusion, and DNN for DDI prediction	[46]
	DeepLGF	PV‐DBOW for obtaining the drug representation vector; GNN encoder; four fusion methods: directly connecting‐based methods, crossing matrix‐based methods, average‐based methods and self‐attention‐based methods	[8]
Neural network‐based	KGNN	GNN, GraphSAGE	[47]
	Eickhoff et al.	The encoder generates plausible negative triplets for the discriminator. The decoder minimizes reconstruction errors. The discriminator is trained to yield a robust KG model. Gumbel‐softmax to resolve gradient disappearance	[40]
	KG2ECapsule	Building graph embedding layer on graph convolution network structure, including message propagation and message aggregation. Three aggregate functions: sum aggregator, concatenation aggregator and neigh aggregator from KGNN [42]	[48]
	DDKG	Encoder is composed of Bi‐LSTM and decoder is composed of LSTM	[49]

### 4.1 Translation‐based KG for DDI

The translation‐based embedding approach uses the distance‐based function (in Euclidean space) to generate embeddings. KG embedding model TransE [41] is proposed based on the principle of translation invariance in the word embedding algorithm word2vec, which predicts existing but non‐corresponding entities and relations from patterns in existing data relations. It takes RELATION as the core and considers the relationships in each triplet as translations from head to tail, which can be simply understood as vector summation. The translation‐based embedding approach can extract the semantic features of drugs from the KG and learn the embedding of entities and relations.

#### 4.1.1 Multi‐scale feature fusion deep learning with MPNN and TransE

To explore the combined effects of drug molecular structure and semantic information in KG, Song et al. [42] proposed a multi‐scale feature fusion deep learning model MUFFIN, which can combine drugs' structural information and KG with rich biomedical information to learn drug representations together. Features from large‐scale KG and drug molecular graphs can be derived through a cross‐layer module to alleviate the limited label data problem. The representation learning module aggregates the neighborhood information of drug nodes through *k* iterations in the message passing phase to update the node representations, then averages the node representations through the readout function to find the final drug representation. The cross‐layer of the feature fusion module first uses cross operation to construct a cross matrix, which expresses the interaction between $u_{i}$ and $v_{j}$ as follows:

$$C_{i}=u_{i}⊗v_{i}=\left[\begin{array}{ccc} u_{i1}v_{i1} & ... & u_{i1}v_{id}\\ ... & ... & ...\\ u_{id}v_{i1} & ... & u_{id}v_{id} \end{array}\right].$$

A CNN with max pooling is then used to learn local interaction features: $a_{i_{l}}=\text{Pooling}\left(\text{RELU}\left(W_{c}*C_{i}+b_{c}\right)\right)$. The global features are used to flatten $a_{i_{l}}=\text{Flatten}\left(C_{i}\right)$. The learned features are then concatenated into a fully connected layer. The scalar layer encodes with the diagonal element product to obtain the diagonal element vectors, which are fed to the fully connected layer to obtain fusion features. The advantage of MUFFIN is that it makes full use of the features extracted from drug molecular structure graphs and the biomedical KG DRKG.

#### 4.1.2 Efficient KG summarization via GNN

GNN can achieve good performance by treating DDI prediction as a link prediction problem, but it ignores biomedical KG, which is beneficial to DDI. To address this issue, Xiao et al. [43] proposed the SumGNN model for multi‐type drug interaction prediction using KGs. SumGNN initializes all entity embeddings with the KG embedding method TransE. Xiao et al. proposed a layer‐independent self‐attention mechanism to generate strength scores for each edge in the subgraph and removed the KG subgraph path with high scores, the self‐attention score is calculated as

$$\alpha_{ij}=\text{Threshold}\left(\varphi \left(\frac{h_{j}^{\left(0\right)}W^{J}{\left(h_{i}^{\left(0\right)}W^{I}+r_{ij}\right)}^{Τ}}{\sqrt{d_{k}}}\right),\gamma \right),$$

where the $W^{J}$ and $W^{I}$ are the self‐attention key weights, $r_{ij}$ encodes the relationship between the entity i and j, $\sqrt{d_{k}}$ is the size of feature vectors for normalization, γ is the signal threshold. Finally, they used multiple channels to aggregate different sets of data sources to improve multi‐type DDI prediction. The advantage of SumGNN is that it effectively anchors correlation subgraphs from KG and leverages extensive external biomedical knowledge to significantly improve multitype DDI predictions.

### 4.2 Tensor factorization‐based KG for DDI

Tensor factorization‐based model treats the KG as a third‐order tensor, and its representative methods include ComplEx [50] and RotatE [51], etc. The ComplEx model is improved based on the DistMult model by introducing complex‐valued vectors, calculating the complex‐valued dot product (Hermitian dot product), and treating its real part as a triplet score. The RotatE model maps entities and relations into a complex vector space and defines each relation as a rotation from the source entity to the target entity, while proposing a self‐adversarial negative sampling technique to generate negative samples based on the current entity and relation embeddings.

#### 4.2.1 KG combined with MHRW and improved neural network TBAN

Since the study of DTI and DDI can help accelerate progress in drug discovery, Zhang et al. [44] combined graph representation learning and neural networks to construct two KGs, KG‐DTI and KG‐DDI. Then two improved graph representation learning models MHRW2Vec and SANA‐ComplEx are used to obtain the feature vectors of drugs and targets. MHRW2Vec includes Metropolis Hasting Random Walk (MHRW) and Word2Vec. MHRW can construct a Markov chain, and it is applied to each node in the KG to generate node sequences by looping. These node sequences are used as the input of the SkipGram (SG) model in Word2Vec. Finally, the obtained feature vectors are fed into an improved neural network model TextCNN‐BiLSTM‐Attention Network (TBAN), which consists of TextCNN, BiLSTM and attention layers, to construct a model MHRW2Vec‐TBAN that can be used for both DTI and DDI prediction. The advantage of MHRW2Ec‐TBAN is that it can improve the node bias problem in traditional random walk algorithms.

#### 4.2.2 Relation‐aware network embedding for drug‐drug interaction prediction

Given that different relationships between drugs may have different effects on drug embedding, Yu et al. [45] proposed a relationship‐aware network embedding model (RANEDDI) for both binary and multi‐relationship DDI prediction by combining multi‐relationship embedding with relation‐aware network structure embedding. RANEDDI regards a drug as a capsule. It first uses the RotatE model to learn the multi‐relationship embedding of each drug entity. The embedding of each entity or relation is denoted as $e_{h}=\left(e_{h}^{\left(1\right)},e_{h}^{\left(2\right)},...,e_{h}^{\left(v\right)}\right)$. The score function of each triplet embedding is defined as: $\text{score}\left(v,r,u\right)=-\|e_{v}\text{○}e_{r}-e_{u}{\|}_{2}^{2}$. RANEDDI then uses the proposed relation‐aware information dissemination mechanism to extract the relation‐aware structure embeddings from the neighbors of each drug entity. At the same time, it learns the embedding of the drug entity and aggregates the embedding of the drug entity with the relation‐aware structure embedding extracted from neighbors to obtain the final embedding of the drug. The embeddings of two drugs are combined to perform binary or multi‐relational DDI prediction according to the prediction task. RANEDDI has the advantage that it can learn a more expressive representation of a drug by combining the two pieces of information contained in a DDI network. But the authors argue that embedding representations cannot be learned from DDI networks when drugs have no neighbors.

#### 4.2.3 Drug knowledge graph (DKG) fusing biochemical information for DDI prediction

Many methods do not consider multi‐scale features and have the limitation of not being able to predict interactions between new drugs. To solve these problems, You et al. [46] built a BioDKG‐DDI model by combining the features of drug molecular structure, drug global features and drug functional similarity. The drug structure is extracted from its molecular structure features by Mol2Context‐vec. Mol2Context‐vec used two‐direction prediction probability. The drug embedding matrix is obtained by integrating multiple atomic embeddings. The drug representation vector is obtained by ComplEx‐DURA to obtain the global features of drugs. The scoring function of ComplEx‐DURA used the inner product similarity:

$$f_{j}^{E}\left(i,k\right)=-\|e_{i}\bar{R_{j}}-e_{k}{\|}_{2}^{2}=2\text{R}\text{e}\left(\langle e_{i}\bar{R_{j}},e_{k}\rangle \right)-\|e_{i}\bar{R_{j}}{\|}_{2}^{2}-\|e_{k}{\|}_{2}^{2},$$

where $e_{i}$ and $e_{k}$ are embedding vectors of entity, $R_{j}$ is a matrix representing relation. To ensure drug specificity and obtain the global features of the drug, the similarity features of the drug are obtained using the Euclidean distance of the four drug‐related receptors, and the four similarity matrices are fused by SNF to obtain integrated matrices. Finally, the features learned from these three modules are fused using the self‐attention mechanism and predicted using a deep neural network. The advantage of BioDKG‐DDI is that it can adaptively learn and integrate three different types of drug features to predict new DDIs.

#### 4.2.4 BKG fusing local–global information to improve the performance of DDIs prediction

Most existing methods only use a single view of information and rarely perform tasks based on biomedical knowledge graphs (BKG). For this reason, You et al. [8] proposed a new deep learning framework DeepLGF. DeepLGF changed Mol2Context‐vec to PV‐DBOW, which can focus well on the order and semantic information of drug symbols by extracting the distributed bag of words version of paragraph vectors. PB‐DBOW utilized Skip‐gram to calculate the vector representation of the whole sequence, the process is as follows:

$$h_{c}=W^{T}x_{c},$$

$$E=\log p\left(v_{0}|v_{c}\right)=u_{0}^{T}h_{c}-\log\left(\sum \exp\left(u_{i}^{T}h_{c}\right)\right),$$

$$E_{\text{sent}}=\log p\left(v_{0}|\text{sen}t_{c}\right)=u_{0}^{T}{h_{c}}^{\prime }-\log\left(\sum \exp\left(u_{i}^{T}{h_{c}}^{\prime }\right)\right),$$

where $x_{c}$ is the one‐hot vector of c‐th center word, $h_{c}$ is the embedding vector of the center word, $h_{c}^{\prime }$ is a sentence embedding vector, E is the loss probability function of the actual context word, $E_{\text{sent}}$ is the probability loss function of the context word, $u_{i}$ is i‐th column of weight matrix W. To better obtain global features of drugs, the BFGNN module based on the GNN encoder‐decoder is constructed to integrate drug similarity feature replacement in the third module. The advantage of DeepLGF is that it fully considers the biochemical information of drugs and KG to extract local‐global information, and it also employs a suitable feature fusion method to enhance features. However, the authors argue that random selection of negative samples will create some noise.

### 4.3 Neural network‐based KG for DDI

Translation‐based embedding and tensor factorization‐based embedding methods such as TransE and CompleEx capture linear relationships between entities using only addition, subtraction or simple multiplication operators, which are simple methods, but they cannot capture the relationships of triples from multiple dimensions, thus these methods have slightly inferior performance in handling multi‐relationship prediction tasks. Using neural networks (such as capsule networks), entity representation can be obtained in a nonlinear form under a specified relational space [48] for multi‐type DDI prediction.

#### 4.3.1 KG with GNN for DDI prediction

Previous methods for DDI prediction based on KGs directly learn the latent embedding vectors of nodes, which limits the acquisition of rich neighborhood information for each entity in KG. To address this limitation, Quan et al. [47] proposed a KG‐based GNN KGNN. KGNN uses GNNs to sample the neighborhood of each node. At the same time, the topological structure information of drug entities in the KG and their semantic association information are considered. The framework consists of three modules: DDI extraction and KG construction, KGNN layer and drug‐drug interaction prediction. The DDI extraction phase uses two datasets to extract drug pairs and the corresponding KG is constructed using the Bio2RDF [52] tool. In KGNN layer, the neighborhood of the entity is sampled, and the method with a fixed‐size neighborhood range is borrowed from GraphSAGE [53]. The embedding representation of the current entity is obtained by aggregating the embedding representation of the entity and the embedding representation of the neighborhood information through three aggregation methods. Finally, the potential representations of drugs and their neighborhood topologies are obtained. The advantage of KGNN is that it can mine associations in the KG to efficiently capture drugs and their potential neighborhoods.

#### 4.3.2 Wasserstein adversarial autoencoder‐based KG embeddings

Existing methods that only apply uniformly random mode to construct negative samples are often too simple and do not effectively train the model, to address these limitations, Eickhoff et al. [40] introduced adversarial self‐encoder AAE to KG for the first time based on Wasserstein distances and Gumbel‐Softmax relaxation [54] and proposed a new framework for KG embedding for DDI prediction tasks. The autoencoder acts as a generator of negative sample triples to generate reasonable negative sample triples for the discriminator. This module solves the gradient vanishing problem with Gumbel‐Softmax after convolution layer and batch normalization. The process of Gumbel‐Softmax can be described as follows:

$$y=\text{one}\_\text{hot}\left(\mathrm{argmax}\left(o_{i}+g_{i}\right)\right),{\hat{\;y}}_{i}=\frac{\exp\left(\left(o_{i}\right)+g_{i}\right)/\tau }{\sum \exp\left(\left(o_{a}\right)+g_{a}\right)/\tau },$$

where $o_{i}$ is the i‐th element of o, $g_{1},...,g_{\left|E\right|}$ are i.i.d. samples drawn from a standard Gumbel distribution, τ is an adjustable parameter referred to as the inverse temperature. Subsequently, the encoder function is refined with the minimum reconstruction error of the decoder. Finally, the generated negative sample triples and the original positive sample triples are fed into the discriminator, which is trained to learn the embedding vectors to form a robust KG model. The advantage of this approach is the introduction of an AAE framework to generate more plausible drugs as negative samples.

#### 4.3.3 Biomedical KG embedding with capsule network

Multiple types of DDI randomly generating negative samples may introduce false negative samples. To address this limitation, You et al. [48] proposed a new framework KG2ECapsule for multi‐label drug interaction prediction based on capsule networks, and integrated a probability‐based negative sampling strategy that can avoid introducing false negative samples. The framework consists of three main components: negative sample construction, graph embedding layer and capsule network layer. High‐quality negative samples are generated first, and then graph embedding layers are constructed, each of which consists of two parts: message propagation and message aggregation. Finally, a two‐layer capsule network is constructed to obtain entity representations in a specific relation space, with K capsules in the first layer and one in the second layer. The strength of KG2ECapsule lies in its ability to produce high‐quality negative samples and the capsule network’s ability to enrich entity representations.

#### 4.3.4 An attention‐based KG representation learning framework

BKGs provides more detailed drug information, to learn more about drugs from KG, Hu et al. proposed an attention‐based learning framework for KG representation DDKG [49] by considering both drug attributes and triple facts in KG, where the KG consists of SMILES sequences of drugs and triple facts combined with other entities. The integrated KG is sent to the encoder‐decoder layer to learn the initial embedding of drugs. Bi‐LSTM is used for the encoder process. The input, forget and output gates of the LSTM are computed as follows:

$$i^{\left(t\right)}=\sigma \left(W_{i}\cdot \left[h^{\left(t-1\right)},e^{\left(t\right)}\right]+b_{i}\right),f^{\left(t\right)}=\sigma \left(W_{f}\cdot \left[h^{\left(t-1\right)},e^{\left(t\right)}\right]+b_{f}\right),o^{\left(t\right)}=\sigma \left(W_{o}\cdot \left[h^{\left(t-1\right)},e^{\left(t\right)}\right]+b_{o}\right),$$

where $i^{\left(t\right)},f^{\left(t\right)},o^{\left(t\right)}\in R^{T},b_{:}\in R^{T}$, and $W_{:}\in R^{\left(d+T\right)\times T}$. Then the neighborhood information is spread to the receiving domain of drug nodes along the top‐ranked network path, and the first‐order neighborhood information is aggregated to learn the comprehensive representation of drugs. Finally, the representations of two drugs are multiplied to estimate their interaction probability. The advantage of DDKG is to make full use of KG information to improve the performance of DDI prediction. However, the authors believe that since the network path used to propagate first‐order neighborhood information is determined by the attention weights of triple facts, it is difficult to obtain the global optimal solution. Multihop attention mechanism or conjoint attention can be considered to solve this problem.

## 5 MULTIMODAL‐BASED METHODS

Multimodal deep learning takes multiple drug features as modalities and combines them for DDI prediction. The drug itself has different features such as target, enzyme and pathway, while the drug also has many different modalities. For example, drugs exist in multiple forms, such as text, molecular graphs, or as nodes in association graphs. Meanwhile, drugs have multi‐view features, such as 1D SMILES, 2D molecular graphs, 3D conformational graphs, etc. We summarize these methods and show them in Table 5.

**TABLE 5:** DDI prediction based on multimodal.

Taxonomy	Model	Drug features	Techniques	Refs.
One‐dimensional drug features‐based methods	DDI‐MDAE	Drug‐drug interactions, substructures, targets, enzymes and pathways	Multimodal deep autoencoders, Jaccard similarity; four operators: Average, Hadamard, L1‐norm and concatenate; RandomForest classifier	[20]
	DDIMDL	Chemical substructures, targets, enzymes and pathways	Jaccard similarity and multimodal deep neural network	[56]
	DANN‐DDI	Targets, enzymes, substructures, and pathways	Deep attention neural network	[57]
	MDF‐SA‐DDI	Substructures, targets and enzymes	Siamese network, CNN, two autoencoders, transformer block with multi‐head attention module. Mixup for better performance	[59]
	DeepDrug	Drug molecular graph and SMILES sequence	RGCN and CNN	[60]
	MLRDA	Chemical structures, drug indications, targets and drug side effects	Single feature representation modules use the autoencoders framework. Aggregation module uses attention mechanism	[61]
Fusion of molecular graph or association graph methods	GNN‐DDI	Enzymes, targets, substructures, and pathways	GNN and Jaccard similarity	[62]
	TP‐DDI	Targets, enzymes, substructures, side effects, offside effects, transporters, pathways, and indications	GCN with self‐attention, SENet for assigning weight to each similarity matrix, and DNN for DDI prediction	[63]
	MADRL	Molecular structure, target, enzyme, pathway, side effect, phenotype, gene, and disease	CUR matrix decomposition, alternating direction method of multipliers (ADMM), graph manifold regularization and GAT	[64]
	Liu et al.	Atom symbol, formal charge, whether the atom is aromatic, its hybridization, chirality, etc	GCN encoder	[65]
	3DGT‐DDI	Drug text features and 3D structural features	MMFF94(RDKit toolkit), SchNet and SCIBERT	[66]
	MIRACLE	Drug 3D molecular structure features and DDI association graph features	Bond‐aware message passing network (BAMPN) in inter‐view. GCN encoder in intra‐view. Mutual information (MI) on the Jensen‐Shannon‐based representation of the KL‐divergence for better performance	[67]
	GoGNN	Drug molecular graph and atoms feature	Convolutional neural network, self‐attention mechanism and graph pooling	[68]
	MDNN	DDI matrix (DKG; target feature, substructure feature and enzyme feature)	GNN for extracting topological structure and semantic relationship, the heterogeneous feature (HF)‐based pathway with Jaccard similarity for heterogeneous feature embedding of drugs	[69]

### 5.1 One‐dimensional drug features‐based methods

Each drug has a variety of different one‐dimensional features, and different drug features have different effects on DDI prediction [20]. Considering multiple features can obtain better DDI prediction performance to some extent. Many existing methods integrate multiple one‐dimensional drug features (such as targets, enzymes, and pathways, etc.) for DDI prediction.

#### 5.1.1 Multi‐modal deep auto‐encoders based drug representation learning method with structural network embedding

Integrating heterogeneous drug features may bring limitations such as heterogeneous properties, non‐linear relations and incomplete data, to alleviate these limitations, Zhang et al. [20] proposed a multimodal depth self‐encoder‐based DDI prediction method DDI‐MDAE, which treats five drug data sources of drug interactions, drug chemical substructures, targets, enzymes, and pathways as five drug feature networks. They use multimodal depth self‐encoder to learn a unified representation of the drug from the five drug feature networks. Finally, a random forest classifier [55] is trained to predict potential DDIs using random forests. The advantage of DDI‐MDAE is that it uses only the structural topology of the drug feature network to learn drug representations, which can significantly improve the performance of sparse networks.

#### 5.1.2 A multimodal deep learning framework for predicting drug‐drug interaction events

To consider more drug features and solve the problem of feature redundancy, Zhang et al. [56] proposed a new method DDIMDL. Zhang et al. collected DDIs from DrugBank database, and extracted 65 categories of DDI events by dependency analysis and event trimming. DDIMDL uses four drug features as input, which are encoded into feature vectors and then the corresponding similarity matrix is calculated by Jaccard similarity. Subsequently, the deep neural network sub‐model is constructed separately for each feature. Using Bottleneck features in the sub‐model can reduce the number of parameters for training, using the ReLU function as the activation function and adding batch normalization between each layer can accelerate convergence. The similarity matrix of each feature is fed into the corresponding neural network and the joint DNN framework is used to combine the sub‐models to learn the cross‐modal representation of drug pairs. The advantage of DDIMDL is to integrate various features by using multi‐modal learning, but the authors believe that the number of DDI events is unbalanced.

#### 5.1.3 Deep attention neural network to enhance DDI prediction

To address how to focus on integrating multiple drug features to predict unknown DDI, Zhang et al. [57] proposed a model DANN‐DDI for enhancing DDI prediction using deep attention neural networks. Attention neural network is used in drug pair feature learning. Zhang et al. collected five types of drug features, and correspondingly constructed multiple drug feature networks to learn a comprehensive representation of drugs. The structured deep network embedding (SDNE) [58] is used to learn the embedding of drug nodes from the constructed drug feature networks. The drug‐drug pair feature learning module integrates drug feature representations learned from the previous five drug feature networks to learn drug pair representations. The attention vector is computed using the ReLU function as the activation function to capture the importance of the drug pair representation in k‐dimensions.

The attention vector is calculated as follows:

$$a_{i,j,k}=\frac{\exp\left({\hat{a}}_{i,j,k}\right)}{\sum_{m=1}^{k}\;\exp\left(a_{i,j,m}\right)},$$

where $a_{i,j}=\left(a_{i,j,1},a_{i,j,2},...,a_{i,j,k}\right)$ is an attention vector, ${\hat{a}}_{i,j}=v^{T}RELU\left(W\left[E_{i},E_{j}\right]+b\right)$, b and W are the bias vector and the weight matrix, $v^{T}$ is the weight vector, k is the dimension of the comprehensive vector. Finally, the learned drug pair representations are fed into a neural network of the same dimensionality for DDI prediction. The advantage of DANN‐DDI is that it can focus on combining different drug information and provide high accuracy performance.

#### 5.1.4 Multi‐source drug fusion, multi‐source feature fusion and transformer self‐attention mechanism

To predict an interaction event between two drugs, rather than just whether there is an interaction, Wei et al. [59] proposed a DDI prediction method MDF‐SA‐DDI based on multi‐source drug fusion, multi‐source feature fusion and transformer attention mechanism. MDF‐SA‐DDI extracts three different drug features and feeds them to four different drug fusion networks, namely Siamese network, CNN and two autoencoders. The potential vectors are subsequently summed to obtain a fifth potential vector. The five potential vectors are connected to obtain new features, which are then fed to the transformer block with the attention mechanism for potential feature fusion. Experimental results with other baseline methods prove that multi‐mode multi‐source feature fusion and data enhancement algorithms can improve DDI prediction performance.

#### 5.1.5 Combining RGCN and CNN for DDI prediction

Due to the lack of systematic evaluation of inherent properties embedded in corresponding chemical structures, Jiang et al. [60] combined RGCN and CNN to propose DeepDrug for DDI prediction, which can be used for interaction prediction of both DDI and DTI. DeepDrug consists of RGCN module, CNN module and combined prediction module. First, the graph and one‐hot representations of each drug are extracted from the SMILE sequence. Then the structural and sequence information of each drug is fed into the RGCN module and the CNN module, respectively, for feature extraction. The RGCN module can learn both node embeddings and edge embeddings by graph convolution. Meanwhile, the convolutional residuals are applied in the GCN module. The representations of each drug learned in the RGCN and CNN blocks are then aggregated using the softmax aggregation function. The aggregated features of the two drugs are then concatenated and sent to the joint prediction module, which is a linear layer with an activation function using the softmax activation function. The strength of DeepDrug is the adoption of a more general graph‐based architecture to facilitate prediction in downstream tasks.

#### 5.1.6 A multi‐task semi‐supervised learning framework for DDI prediction

Since the sparsity of DDI labels increases the risk of overfitting multi‐task learning models when combining multiple drug features, to address this issue, Wang et al. [61] integrated multiple drug features combined with multi‐task learning to construct a new semi‐supervised learning framework, MLRDA. It includes *n* Single Feature Representation Modules (SFRM) and an Aggregation Module. Each of *n* drug features is used as input to *n* SFRMs, and *n* binary vectors are output. The *n* outputs are aggregated in the aggregation module to get the final output. SFRM adopts an automatic encoder framework, including h+1 layers. The first h/2+1 hidden layers are encoders to learn the representation of each input, and the last h/2 layers are decoders to reconstruct the input. The encoder aims to divide the input into two parts at layer h/2+1: factor $\hat{Y}=\left\{{\hat{y}}_{1},{\hat{y}}_{2},...,{\hat{y}}_{i}\right\}$ related to the DDI prediction and factor *Z* unrelated to the DDI prediction, are then encoded, where the learning of ${\hat{y}}_{i}$ can be considered as a binary classification task, and each SFRM formulates the DDI problem as a multi‐label classification problem. The advantage of MLRDA is the introduction of CuXCov, thus effectively utilizing labeled and unlabeled drug data that are beneficial for DDI prediction.

### 5.2 Fusion of molecular graph or association graph methods

Drugs exist in many forms, such as nodes in molecular graphs or association graphs. At the same time, it also has multi‐view features, such as 1D SMILES, 2D molecular graphs, 3D conformation graphs. The performance of DDI prediction can be improved by the simultaneous use of multiple drug forms. For example, the simultaneous use of drug molecular graphs and drug association graphs, and the simultaneous use of drug features and DKGs.

#### 5.2.1 Generating drug embeddings from attribute heterogeneous networks for DDI prediction using GNN models

Drug features may be correlated and contain redundant information that makes it challenging to effectively integrate the various features, to alleviate this limitation, Lakizadeh et al. [62] performed DDI prediction in two stages and proposed a deep learning model GNN‐DDI using GNN to integrate multiple drug features. First, the similarity matrices are constructed using four different drug feature matrices, and then the attribute heterogeneous networks are constructed using the DDI graph and the four similarity matrices, with one similarity matrix used as the node attribute in each step. Attribute heterogeneous network embedding can provide better drug representation in different drug interaction types. The recent algorithm [70] is used to learn the embeddings of attribute heterogeneous networks and generate four drug embedding matrices. Subsequently, the drug embedding matrices are concatenated to obtain one‐dimensional feature vectors, which are fed into the fully connected layer for DDI prediction. The advantage of GNN‐DDI is that it uses attribute heterogeneous network embeddings of different drug interaction types to provide better drug representations.

#### 5.2.2 Multi‐modal with two‐pathway deep neural network

Since there are many factors that can cause DDI, Wang et al. [63] considered several factors and designed a dual pathway drug‐drug interaction framework TP‐DDI for DDI prediction using multimodal data. The model consists of two modules, topology‐based pathway and similarity‐based pathway. The topology‐based pathway uses GCN and self‐attention mechanism to learn drug embeddings from the drug molecular substructure graph, uses GCN and ReLU function to learn interaction relationship embeddings from the DDI network, and uses multi‐head attention mechanism to extract drug topology. Similarity‐based pathways use multiple drug features and feature similarity matrices to generate feature representation. Finally, the deep neural network is used to concatenate the latent feature vectors of a pair of drugs and calculate the probability score of DDI. The advantage of TP‐DDI is that it effectively utilizes the joint representation learning of topology information and similarity information for efficient DDI prediction. However, the authors believe that some of the new predicted DDIs do not exist in the database and require further biological validation.

#### 5.2.3 Multi‐attribute discriminative representation learning for prediction of adverse DDI

The existing models ignore the consistent and unique properties among attributes, to solve this limitation, Liu et al. [64] proposed a new model MADRL. They used CUR matrix decomposition to select features for each attribute matrix, which is achieved by minimizing the following equation:

$$\min_{p^{m},q^{m},{\hat{U}}^{m}}{\left\|X^{m}-X^{m}D\left(q^{m}\right){\hat{U}}^{m}\text{diag}\left(p^{m}\right)X^{m}\right\|}_{F}^{2}$$

$$s.t.\;l_{K}^{T}p^{m}=\bar{k},p^{m}\in {\left\{0,1\right\}}^{k},$$

$$l_{C_{m}}^{T}q^{m}={\bar{C}}_{m},\theta^{m}\in {\left\{0,1\right\}}^{C_{m}},$$

where $D\left(p^{m}\right)$ returns a diagonal matrix with $p^{m}$ being its diagonal elements, $l_{k}$ is an k‐dimensional all one vector, $p^{m}$ and $q^{m}$ are two indicators to respectively present whether a drug and a feature is selected or not. Liu et al. transformed the above equations into a convex optimization problem by combining the alternating direction method of multipliers (ADMM) [71]. Moreover, the similarity between two pairs of drug attributes is calculated and introduced into the attribute representation reconstruction in the form of a graph structure as a regularization term. Based on the selected drugs and features, the reconstructed representations of the adverse drug pair features are then derived. They also designed a GAN framework to learn the common and specific attribute representations of each adverse drug pair and merge these drug pairs into multi‐attribute ADDI predictions. The strength of MADRL is its compatibility with any type of input attribute and its ability to explore these input attributes’ respective effects on ADDI predictions.

#### 5.2.4 Multi‐type feature fusion based on GNN

Some methods consider only single information of the drug, which is deficient in terms of robustness and scalability, to alleviate this limitation, Liu et al. [65] proposed a new DDI prediction method MFFGNN based on GNNs by fusing topological information from molecular graphs and proposed a graph feature extraction module (MGFEM). The model takes SMILES sequences and molecular graphs as input, and then extracts multiple drug features through the molecular graph feature extraction module MGFEM and the SMILES sequence feature extraction module SSFEM, respectively. Multiple drug features are then aggregated to obtain intra‐drug features, which are fed into the multimodal feature fusion module to fuse drug features. The updated drug features are obtained using a GCN encoder that sets a gating mechanism to control how much neighborhood information is passed to the nodes and finally enters the MLP for DDI prediction. The advantage of MFFGNN lies in the efficient fusion of information from drug molecular graphs, SMILES sequences, and DDI graphs to extract global and local features in molecular graphs.

#### 5.2.5 Combination of 3D GNN and pre‐trained text attention mechanism

Many existing methods ignore the 3D structure information of drug molecules and do not fully consider the contribution of molecular substructure in DDI. To solve this problem, Chen et al. [66] proposed a new DDI prediction method 3DGT‐DDI by combining textual description information and molecular 3D structure features, which consists of a 3D GNN and textual attention mechanism together. The feature extraction model SCIBERT is used to extract text information, and the location of drug names is extracted and embedded to enhance the feature extraction ability of the context. In addition, the feature weight visualization of text and molecular graph structure could prove the interpretability of the model. To obtain 3D structures of two drug molecules using SchNet [72] for geometric modeling of position information and types of atoms in 3D graph model. The general 3D graph model message passing update equations are as follows:

$$e_{k}^{\prime }=\phi^{e}\left(e_{k},v_{r_{k}},v_{s_{k}},E_{s_{k}},u,\rho^{p\to e}\right),$$

$$v_{i}^{\prime }=\phi^{v}\left(v_{i},\rho^{e\to v}\left(E_{i}\right),\rho^{p\to v}\right),$$

$$u^{\prime }=\phi^{u}\left(\rho^{e\to u}\left(E^{\prime }\right),\rho^{v\to u}\left(V^{\prime }\right),\rho^{p\to u}\right),$$

where ϕ is the update function for the same class of features, ρ is the transfer function from one class of features to another, $\rho^{p\to e}$ is used to get the global feature and the feature from the union, $\rho^{p\to v}$ is used to aggregate the features of edges. The text information is extracted with SCIBERT, the text feature extraction model is based on BERT [73], and the location embedding of the drug name is extracted to enhance the feature extraction ability of the context, thus enhancing the prediction ability of the model for DDI. The advantage of 3DGT‐DDI is that it combines 3D structural features and position features of drug entities to improve the prediction effect.

#### 5.2.6 Multi‐view graph contrastive representation learning based on BAMPN and GCN encoder

Existing methods are often limited to the use of inter‐view drug molecular structures, while ignoring the intra‐view interactions of drugs. To address this issue, Wu et al. [67] proposed a new method MIRACLE. They regarded the DDI network as a multi‐view graph, capturing both inter‐view molecular structure and intra‐view molecular interactions, and proposed a new unsupervised contrastive learning component to balance and integrate features learned from different views. In inter‐view, the drug molecular graph is encoded as drug embedding through the bond aware message passing network (BAMPN). In intra‐view, external DDI relationships are integrated into drug embeddings using GCN encoders. The GCN encoder framework is as follows:

$$D^{\left(1\right)}=U\left(A,G,W_{u}^{\left(0\right)},W_{u}^{\left(1\right)}\right)=\hat{A}\text{ReLU}\left(\hat{A}GW_{u}^{\left(0\right)}\right)W_{u}^{\left(1\right)},$$

where $W_{u}^{\left(0\right)}$ and $W_{u}^{\left(1\right)}$ are two learnable weight parameters at the 0‐th and 1‐st layer of the GCN encoder, A and G are the adjacency matrix and the attribute matrix of DDI networks, respectively. Graph contrastive learning‐based methods are used to balance information from different perspectives to integrate multi‐view drug representation vectors to update drug embeddings. In addition, the Jensen‐Shannon‐based [74] mutual information (MI) estimator is used on intra‐view and inter‐view. The advantage of MIRACLE is that using the attention mechanism to learn inter‐view drug representation vectors can automatically select the most important atoms in DDIs, while ignoring some noisy and meaningless substructures. At the same time, it can learn more comprehensive vectors of drug representations by integrating multi‐view information. DSN‐DDI [75] also uses two views, intra‐view and inter‐view. The intra‐view consists of a single‐drug graph, while the inter‐view involves a bipartite graph. In the intra‐view, nodes representing drugs within a single drug graph are updated by aggregating information from their neighbors within the same drug. In the inter‐view, the nodes of one drug are updated by aggregating information from all nodes of the other drug.

#### 5.2.7 A graph of graphs neural network GoGNN

Existing work on structured entity interaction prediction cannot properly utilize the unique graph model in the graph. To address this problem, Lian et al. [68] first applied GNNs to Graph of Graphs and proposed GoGNN. In the molecular graph $G_{M}$, the nodes $\left\{a_{i}\right\}$ represent atoms and the edges $\left\{e_{ij}\right\}$ represent the bonds between atoms $a_{i}$ and $a_{j}$. In the global interaction graph $G_{I}=\left\{N,E_{I}\right\}$, N is the number of nodes, that is, the number of $G_{M}$, and $E_{I}$ is the interaction edges between the molecular graph $G_{M}$. In the DDI dataset, each edge also has an attribute vector $e^{r}$. GoGNN uses GCNs in molecular GNNs, using the self‐attention mechanism in each layer to obtain the self‐attention score $s_{l}$ for molecule graph $G_{M}$ with n atoms at $l^{\text{th}}$ layer:

$$s_{l}=\sigma \left({\bar{D}}^{-\frac{1}{2}}\bar{A}{\bar{D}}^{-\frac{1}{2}}M_{l}W_{\text{att}}^{l}\right),$$

where $W_{\text{att}}^{l}$ is the attention weight matrix, $\bar{A}$ is the adjacency matrix, $\bar{D}$ is the diagonal degree matrix of $\bar{A}$. Then the graph pooling layer finds the top‐⌈γn⌉ atoms with the highest attention scores. Finally, the output of the graph pooling layer is concatenated to obtain the entire $G_{M}$ representation $X_{G_{M}}$. GAT is used on the interaction GNN $G_{I}$, and the information of edges is considered when aggregating. In prediction, the DDI and CCI tasks are regarded as linked prediction problems. The advantage of GoGNN is that it leverages the dual mechanism in the view of graph of graphs to capture information in entity graphs and entity interaction graphs hierarchically.

#### 5.2.8 A multimodal deep neural network for DDI events prediction

Most existing approaches pay little attention to potential correlations between DDI events and other multi‐modal data, such as targets and enzymes. To solve this problem, Gao et al. [69] designed a two‐pathway framework MDNN to obtain multimodal representations of drugs, including DKG based pathway and heterogeneous feature (HF) based pathway. DKG uses GNN to extract the topological structure and semantic relationships between drugs on the constructed KG. The semantics feature score between drug $d_{i}$ and tail entity $r_{in}$ is computed as follows:

$$\pi_{d_{i,r_{\text{in}}}}^{\left(l\right)}=\text{sum}\left[\left(e_{d_{i}}^{\left(l-1\right)}{}^{\circ}e_{r_{\text{in}}}^{\left(l-1\right)}\right)W_{1}^{\left(p\right)}+b_{1}^{\left(p\right)}\right],$$

where $e_{r_{\text{in}}}^{\left(l-1\right)}$ is the relation representation between drugs, $e_{d_{i}}^{\left(l-1\right)}$ is the drug $d_{i}$ representation generated from the previous message passing steps, $W_{1}^{\left(p\right)}$ is the trainable weight matrix, $b_{1}^{\left(p\right)}$ is the bias vector and p is the number of fully connected layers. HF is used to extract drug information from different heterogeneous features. Finally, the information learned from two channels is fused to obtain the complementary relationship between the multimodal representations of the drugs through the multi‐modal fusion neural network layers for DDI prediction. The advantage of MDNN is that it effectively utilizes topological information and semantic structure, and effectively explores the cross‐modal complementarity of multimodal data.

## 6 CHALLENGES AND OPPORTUNITIES

Though there has been a great improvement in DDI prediction using deep learning, this field still faces many limitations and problems that provide more opportunities and challenges for future DDI prediction research. Therefore, we summarize the following points.

### 6.1 Data sparsity

The training of deep learning models usually relies on a large amount of high‐quality labeled data. However, in practical applications, the original dataset usually faces problems such as insufficient data and noisy information, which usually negatively affect prediction accuracy. Most methods treat the unlabeled data in the original dataset as negative samples, but there are still positive samples of data in the unlabeled data. The idea of adversarial learning can be introduced to generate high quality and reasonable negative samples to transform the dataset.

Meanwhile, the constructed DDI network usually faces network sparsity problem. When learning embeddings from the network, the current drug cannot be processed if it has no neighbors. For the network sparsity problem, other biological networks can be used as complementary data sources, such as drug‐protein networks and information aggregation can be performed in these networks.

### 6.2 Rare DDI events

Rare DDI events are some of the DDI events with extremely low possibilities of occurrence. Most models do not perform well on rare DDI datasets. Small sample learning can be introduced to handle rare DDI. However, in some events of few‐shot learning, there may be only one instance in an event, causing this instance to be classified only to the support set *S* or query set *Q*, which cannot be classified in detail. At this point, the introduction of zero‐shot learning can be considered to alleviate this problem.

### 6.3 Interpretability

Although deep learning has been widely applied in many fields, many deep learning models are still black‐box models and lack interpretable DDI prediction models. Predictive DDI models with interpretability are important for medical practitioners such as physicians to jointly dispense drugs to avoid adverse effects on patients.

One approach to enhance interpretability is molecular structure optimization to avoid serious adverse reactions. First, substructures with important properties are found from existing molecular structures, and then these substructures are refined to design new molecules with better properties. Extraction of important subgraphs from molecular graphs based on molecular properties for DDI prediction.

### 6.4 3D molecular representation

Many DDI prediction models use only the two‐dimensional structure of drugs (e.g., traditional SMILES) as input, which cannot learn a comprehensive drug representation and may result in excessive memory and time consumption during data processing. However, 3D structural features of drugs often have richer information than 2D structures, and this information facilitates DDI prediction.

Therefore, we propose that future work can make more use of the spatial structure of drugs, such as fusing 2D topological structures information with 3D conformation information to jointly learn the drug representations to obtain a more comprehensive drug representation for more accurate DDI prediction.

### 6.5 Multi‐view feature learning

Drug molecules often contain multiple atomic and bond features and structural information within them, as well as multiple potential interactions. Therefore, capturing interactions within the molecular view of the drug is also crucial for DDI prediction. Combining both intra‐view and inter‐view interaction patterns of drug molecules and learning embedding representations of drugs using multiple views enables fuller utilization of the rich information in the DDI network. In addition, large‐scale language models (LLMs) have demonstrated promising outcomes across various domains. Employing LLMs (such as BioBERT [76]) for pre‐training drug‐related text descriptions can learn the representation of drugs from another perspective, which is helpful for DDI prediction.

## AUTHOR CONTRIBUTIONS

**Xinyue Li:** Investigation; methodology; writing – original draft and editing. **Zhankun Xiong**: Investigation; methodology; writing – review and editing. **Wen Zhang**: Conceptualization; supervision. **Shichao Liu**: Conceptualization; funding acquisition; supervision.

## CONFLICT OF INTEREST STATEMENT

The authors Xinyue Li, Zhankun Xiong, Wen Zhang and Shichao Liu declare that they have no conflict of interest or financial conflicts to disclose.

## ETHICS STATEMENT

This manuscript does not involve a research protocol requiring approval by the relevant institutional review board or ethics committee.
